# Patterns of Sun Protection Behaviours among Australian Adolescents and Adults over a Six-Year Period

**DOI:** 10.3390/curroncol30080520

**Published:** 2023-07-26

**Authors:** Karlijn Thoonen, Sade Woodhouse, Carolyn Minto, Sally Blane, Zenobia Talati

**Affiliations:** 1 Melbourne Centre for Behaviour Change, Melbourne School of Psychological Sciences, The University of Melbourne, Melbourne 3010, Australia; 2 School of Population Health, Curtin University, Perth 6845, Australiazenobia.talati@curtin.edu.au (Z.T.); 3 Cancer Council Western Australia, Perth 6008, Australia; sally.blane@cancerwa.asn.au

**Keywords:** skin cancer prevention, sun protection, adolescents, repeated cross-sectional design

## Abstract

The major cause for skin cancer is the excessive and unprotected exposure to ultraviolet radiation (UVR), which can be prevented by engaging in sun protection behaviours. As longitudinal studies on both adolescents’ and adults’ performances of sun protection behaviours are limited, the current study aimed to investigate changes in sun protection in these population segments in Western Australia, a region with high annual UVR. During six summer seasons (2015/16 to 2020/21), cross-sectional surveys were conducted among 1806 adolescents (14 to 17 years old) and 1808 adults (18–45 years old), investigating the frequency of five sun protection behaviours (wearing clothing, applying sunscreen, wearing a hat, wearing sunglasses, and seeking shade) and sun avoidance (staying indoors). Over the six-year period, staying indoors increased in both groups. Among adolescents, a decrease in wearing clothing and sunglasses and an increase in seeking shade was demonstrated, and hat and sunscreen use remained relatively stable. Among adults, an increase in sunscreen use was shown, whereas all other sun-related behaviours remained consistent over the six-year period. The results from this study can provide directions for health communications focusing on improving sun protection behaviours among both adolescent and adult populations.

## 1. Introduction

Skin cancer, consisting of melanoma and non-melanoma skin cancer (NMSC) is the most common cancer globally, and incidence rates have been increasing rapidly over the last several decades [1,2]. Australia is among the countries with the highest incidence, and it has been predicted that two-thirds of Australians will be diagnosed with skin cancer during their lives [3]. Early detection and treatment are costly to the Australian health system, with a healthcare expenditure of over AUD 259 million for melanoma and AUD 1.46 billion on NMSC in 2019 [4].

Risk factors for skin cancer include unprotected exposure to ultraviolet radiation (UVR), a history of several sunburns, and sensitive skin [5,6]. Therefore, skin cancer is considered a highly preventable malignancy [7,8]. Recommended sun safety strategies to protect the skin consist of five behaviours (i.e., wearing protective clothing, a wide-brimmed hat, and sunglasses, applying sunscreen with a sun protection factor (SPF) of >30, and seeking shade [9,10,11]). Additionally, staying indoors during peak UVR hours has been measured as a sun-avoidance strategy [12]. These prevention efforts are especially important in Western Australia (WA), where UVR levels reach 11 on average during summer [13].

Sun protection during childhood and adolescence have been marked as particularly important since skin damage in these stages of life are strongly related to skin cancer risk in adulthood [14,15]. Although important, studies have revealed that adolescents’ sun protection behaviours are generally poor [16,17]. From an Australian perspective, research has shown that sun protection strategies in adolescents seem to be inconsistent. For example, hat use has been reported less frequently in the last three decades, whereas wearing clothing and sunscreen appeared to remain relatively stable [18,19]. Sunglasses were frequently worn from 2006 to 2008 but returned to the baseline levels in 2012 and continued to decline in 2016 [18]. Sun avoidance seemed to increase from the early 2000s [18,20,21]. Additionally, sunburn incidence seemed to be consistently prevalent, with between 21 and 25% of adolescents in the area of Melbourne reporting a sunburn during summer between 2003-04 and 2016-17 [21,22,23].

Patterns in sun protection behaviours among Australian adults have yielded more positive results. For example, studies from Melbourne showed that the use of sunscreen along with one or more other methods of sun protection increased between the late 1980s and 2010s. This resulted in a decrease in the proportion of unprotected skin until the late 1990s, and rates of sunburn declined until 2002 [24,25]. Nevertheless, inconsistencies were also observed. For example, findings of wearing protective clothing were inconsistent across studies [12,20] and hat wearing seemed to decrease between the summer season of 2003/04 and 2010/11 [21]. A recent observational study reported that older adults and women consistently performed sun protection behaviours more frequently than adolescents and younger adults [26]. These fluctuations highlight that sun protection behaviours are prone to change and that public health efforts are needed continuously and systematically [25,27].

Adolescence marks an important developmental phase in which normative beliefs are changing and shifts take place from parental impact towards peer influences [11]. Self-consciousness and the internalisation of norms and values increase, making it a crucial period for the formation of habitual behaviours [28,29]. From a sun protection perspective, studies have demonstrated that the transitioning phase from childhood to adolescence is especially important for independently learning and performing sun safety strategies [17,30]. Moreover, appearance-based norms become more important in adolescence, including the desire for a tan and tanning behaviours [31,32,33], and sun protection attitudes seem to decline [16].

Australia has established skin-cancer-prevention efforts that have been ongoing for decades, consisting of various public health interventions focusing on improving skin cancer knowledge and sun safety behaviours on both a state and national level [27]. The most widely recognised is Cancer Council Victoria’s major skin-cancer-prevention campaign ‘Slip, Slop, Slap’, that was first broadcasted in the 1980s [34]. The campaign’s effects had long-lasting success, with a dramatic improvement seen in Australian adults’ sun protection behaviours up until the early 2000s [20,24]. Besides individually focused successes, policy and institutional changes were also established [27].

By updating current knowledge on sun protection behaviours of two important population segments, directions for improvement and tailored content for skin-cancer-prevention efforts can potentially be provided. As previously repeated cross-sectional survey studies between 2001 and 2016 demonstrated little to no improvement in adolescent sun-related behaviours while adult behaviours seemed to remain relatively stable [12,18,20], this study aimed to gain insight in Western Australian adolescent and adult sun-related behaviours over a more recent six-year period (2015/16 and 2020/21).

## 2. Materials and Methods

### 2.1. Participants and Recruitment

A telephone-based survey study was conducted among adolescents and adults, using a repeated cross-sectional design over six summer waves (2015/16–2020/21). This study was part of a larger sun protection monitoring and campaign evaluation study of Cancer Council WA. Of interest for this study were adolescents aged between 14 and 17 and adults aged between 18 and 45, residing in WA. In total, 3614 respondents answered a telephone survey, consisting of 1806 adolescents and 1808 adults.

Quotas were set each year to recruit approximately 300 respondents and ensure the sample consisted of 50% males and 50% females, and 50% adolescents and 50% adults. Quotas were also set by location with the aim of recruiting 30% regional residents. From 2015/16–2017/18 the Electronic White Pages database was used for randomly selecting and calling households in WA, and from 2018/19 onward, the Australian Residential Database was used. All data collection took place during January and February (Australian peak summer). Table 1 shows the key sample demographics.

### 2.2. Measures

A sun protection survey was administered via computer-assisted telephone interviewing (CATI) to assess respondents’ attitudes towards being outside in the sun and their engagement in six sun-related behaviours. Demographic characteristics were assessed by asking about respondents’ age (open question), gender (1 = male, 2 = female), skin type, by asking about the skin colour without a tan (1. very fair, 2. fair, 3. medium, 4. olive, 5. dark, 6. very dark, 7. black, 8. don’t know/can’t say), and area of residency by asking respondents about their postcode and suburb. Five behaviours comprised outdoor sun protection behaviours (wearing protective clothing, wearing sunscreen, wearing a wide-brimmed hat, wearing sunglasses, and seeking shade), and the sixth concerned total sun avoidance (staying indoors). Respondents were asked to indicate how often they would engage in the sun-related behaviours when being outside on a summer day for more than one hour between peak UV hours (between 10 a.m. and 3 p.m.) on a 5-point Likert scale (1 = never, 2 = rarely, 3 = sometimes, 4 = usually, 5 = always).

### 2.3. Procedure

This study was approved by a University Human Ethics Committee. Potentially eligible households were approached by telephone, after which a trained interviewer asked whether they could speak to a household member aged between 14 and 45 years and explained the study’s interest in attitudes about the sun protection of WA residents. Most survey items were based on a structured interview guide with fixed responses for the interviewer to choose from. After finalising the survey, the interviewer thanked the respondent and provided their name, affiliation, and contact information for the respondent to use when questions would arise. Households without members aged between 14 and 45 were recorded as ineligible. Informed consent was obtained when a respondent agreed to continue with the survey. Parental consent was required for minors (aged < 16 years) to participate.

### 2.4. Statistical Analyses

Descriptive statistics were computed to explore the data and verify normal distribution of gender and age. No missing values were present except for ‘Don’t know/Can’t say’ responses on the skin type question, that were treated as missing values.

The dependent variables consisted of composite scores of five reported sun protection behaviours and sun avoidance, and the independent variables consisted of the survey year. Pearson’s chi-squared tests of independence were performed to compare the mean responses for each sun-related behaviour per survey year, stratified by age group (adolescent or adult) [35]. Post hoc Z-tests of proportion with Bonferroni correction were conducted to identify significant differences [36]. Based on these results, one hierarchical linear regression analysis per age group was performed to test for associations between survey year and sample means of six sun-related behaviours, after controlling for demographic variables (i.e., age, gender, skin type, and area of residency). Effect sizes were examined using Cramer’s V (φ), considering Cohen’s suggestion of effect sizes (i.e., 0.10 = small, 0.30 = medium, and 0.50 = large) [37,38]. An alpha level of *p* < 0.05 was predefined to determine statistical significance. All data analyses were performed using IBM SPSS Statistics.

## 3. Results

Information on all frequencies of engagement in sun-related behaviours of adolescents and adults per survey year can be found in Table 2. Consider Table 3 for sample means stratified by survey year and age group.

### 3.1. Adolescents

Wearing sunscreen was the most frequently reported sun protection behaviour in adolescents across all survey years (M = 3.65), followed by seeking shade (M = 3.39), and hat wearing (M = 3.04). Adolescents most often reported wearing protective clothing rarely to sometimes (M = 2.80). Wearing sunglasses was the least frequently reported sun protection method across all survey years (M = 2.64).

The proportion of adolescents that reported ‘rarely’ wearing protective clothing increased by almost 90% from 2015/2016 to 2020/21, and the number of respondents that stated ‘always’ wearing protective clothing decreased by more than 60% from 2015/16 to 2018/19. Adolescents who reported that they ‘rarely’ sought shade decreased by almost 60% between 2018/19 and 2019/20. The proportion of adolescents that reported to ‘never’ wear sunglasses increased by almost 66% between 2017/18 and 2020/21. Adolescents that reported ‘never’ for staying indoors decreased from 2016 and all subsequent years, and the proportion of respondents reporting that they ‘usually’ stay inside increased by almost 41% from 2016/17 to 2020/21. Small associations were found between survey year and the frequency of wearing protective clothing (φ = 0.18), seeking shade (φ = 0.14), wearing sunglasses (φ= 0.20), and staying indoors (φ = 0.18).

Results from the linear regressions to determine predicting factors for engaging in sun-related behaviours are presented in Table 4. In the first model where survey year was not included, skin type was negatively associated with the frequency of reported sun-related behaviours (*p* < 0.001). When adding survey year and controlling for age, gender, skin type, and area of residency, results showed that skin type was the only significant remaining factor explaining the variance in outdoor sun protection behaviours (*p* < 0.001).

### 3.2. Adults

Adults reported wearing sunglasses most often across all survey years (M = 4.17), followed by using sunscreen (M = 3.77). The least reported sun protection behaviour was wearing protective clothing (M = 2.92).

The proportion of adults that reported to ‘never’ wear sunscreen decreased by 68.4% between 2015/16 and 2017/18, and ‘always’ wearing sunscreen was reported more frequently in 2016/17 and 2018/19 compared to other survey years. The proportion of adults that reported to ‘never’ stay inside decreased by 77.3% from 2016/17 to 2019/20, and ‘rarely’ staying inside was reported 52.2% less often from 2016/17 to 2019/2020. Associations between survey year and the frequency of using sunscreen and staying indoors were found (φ = 0.16 and φ = 0.18, respectively).

In the first regression model, the predictive values for age, gender, skin type, and area of residency were tested. The results showed that age was positively associated (*p* < 0.001) and skin type was negatively associated (*p* < 0.001) with the frequency of reported sun protection behaviours. When controlling for age, gender, skin type, and area of residency in the second model, age (*p* < 0.001) and skin type (*p* < 0.001) remained significantly associated with adult sun protection behaviours. These results can be found in Table 4.

## 4. Discussion

This study examined the reported performance of recommended sun protection strategies by Australian adolescents and adults over six summers (2015/16 to 2020/21). The insights provided through these observations could potentially assist directing future skin-cancer-prevention campaigns. The findings indicated that adolescent sun protection behaviours are suboptimal and have not improved during the study period. Extending on a previous study among WA adolescents, most behaviours remained relatively consistent [18]. Adults seemed to perform sun protection strategies regularly and their behaviours show a relative consistency throughout the six-year period, which is comparable to the behaviours reported over an earlier time period [12].

The finding that adolescents seem to engage less in sun protection behaviours than adults replicate other studies conducted in Australia [23], as well as in the United States [31], Germany [38], and Ireland [39]. More specifically, the least performed and declining sun protection behaviour among adolescents was wearing sunglasses. This result extends previous cross-sectional research in Australia, reporting an increase in adolescent sunglasses usage from 2001/02 to 2010/11 with a steep drop in 2012 [18]. Another study from Australia found a decrease in sunglasses use among adolescents as well [20]. This is significant given that 50% of total UV exposure up to age 60 occurs before age 20 [40], making adolescence a critical period for determining future skin cancer and eye damage risk. More specifically, young people are particularly vulnerable to UVR because they have larger pupils and more transparent ocular media, making the use of eye protection from an early age of considerable importance [41].

Previous research among adolescents suggested that the desirability of a tan, having a non-favourable attitude about sun protection and a strong influence of fashion trends on wearing protective garments might explain poor sun protection compliance [42,43,44]. Moreover, an absence of long-term risk perceptions regarding skin cancer and a future quality of life among adolescents might function as a barrier for sun protection performance [45]. An Australian study aimed at understanding adolescent sun protection attitudes and behaviours found several misperceptions (e.g., about the risks of unprotected sun exposure and when to apply sunscreen) and a strong desire for a tanned skin, which persisted when adolescents were aware of the health-risks involved [46]. As several determinants (e.g., skin cancer knowledge, skin cancer risk perceptions, sun protection attitudes) and sun-exposure habits influence actual sun protection behaviours, it could be relevant to further investigate this relation among these population segments as well [20,42,46]. Based on the findings from this study, it may be worth further exploring emotions evoking skin-cancer-prevention campaigns as this could be a potential facilitator for positive change. For example, emphasising more immediate outcomes instead of long-term risks such as avoiding sunburn as a motivator for sun protection instead of decreasing skin cancer risk might be beneficial [45,46].

Wearing protective clothing was the second lowest reported sun protection method among adolescents and the least popular behaviour among adults, which is concerning. Covering the skin with clothing has been proposed as an important sun protection strategy and might potentially be more effective than hat-wearing or sunscreen use in preventing skin damage [23,47,48,49]. Since clothing is subject to changes in fashion and protective clothing might be perceived as uncomfortable or inconvenient, demonstrations of role models that are covering up with comfortable and wearable clothing might be effective in sun protection campaigns [49,50,51]. Furthermore, sun avoidance increased among both adolescents and adults. While staying indoors is favourable since it may reduce skin cancer risk, it is debatable whether this is a favourable tendency considering the increase in screen time that has been observed among Australian children and adolescents since 2012, which has been associated with less engagement in physical activity [52].

Mobile apps may provide an effective strategy for disseminating tailored sun protection messages to adolescents [53,54]. Moreover, social media sites such as Twitter have the potential to send health communications to large population groups, and adolescents seem to intentionally use social media for seeking specific health information [55,56]. An Australian study has shown that Twitter is being used for sharing sun protection experiences and advice, especially during days with high temperatures [57]. Utilising social media for prompting the use of sun protection could potentially be an effective strategy for enhancing adolescent sun protection behaviours.

## 5. Limitations

There are a few limitations in this study that are worth mentioning. First, this study used a repeated cross-sectional study design, meaning that each time point included a different sample of respondents and that the findings represent changes at a population rather than an individual level [58]. Second, this study relied on self-reported behaviours, allowing for the possibility of a recall bias and respondents providing socially desirable answers [59,60]. To minimise this, outliers in the data were carefully examined and standard deviations and confidence intervals were reported. Third, self-reported sunscreen use has shown to correspond with objective sunscreen use in previous research [59]. Finally, this study did not assess factors that may relate to or influence sun-related behaviours (e.g., sun exposure, prior sun protection knowledge and exposure to sun safety interventions). Future studies should ideally include these factors to investigate the underlying components of engagement in sun protection behaviours.

## 6. Conclusions

This study aimed to examine changes in sun-related behaviours of both adolescents and adults in Western Australia over a six-year period (2015/16 to 2020/21). Adolescents reported engaging less in sun protection behaviours than adults, and decreases were found in wearing sunglasses among adolescents. Wearing protective clothing was among the least reported behaviours of both adolescents and adults. Staying indoors increased over the six-year period among both groups. The findings from this study contribute to current insights of adult and adolescent sun-related behaviours and can potentially assist in shaping future health communications by tailoring message content.

## Figures and Tables

**Table 1:** Sample demographics (*N =* 3614).

Characteristic	Adolescents *n* = 1806		Adults *n* = 1808	
	*n*	%	*n*	%
Age				
14–15	885	49.0		
16–17	921	51.0		
18–24			606	33.5
25–35			606	33.5
36–45			596	33.0
Gender				
Male	906	50.2	903	49.9
Female	900	49.8	905	50.1
Skin colour				
Very fair	222	12.3	264	14.6
Fair	591	32.7	641	35.5
Medium	501	27.7	489	27.0
Olive	363	20.1	344	19.0
Dark	101	5.6	61	3.4
Very dark	11	0.6	5	0.3
Black	17	0.9	4	0.2
Location				
Metropolitan	1277	70.7	1245	68.9
Regional	529	29.3	563	31.1

**Table 2:** Frequencies of engagement in sun-related behaviours per survey year for adolescents and adults.

Variables	Survey Year											
	2015/16		2016/17		2017/18		2018/19		2019/20		2020/21	
Wear protective clothing	Adolescents	Adults	Adolescents	Adults	Adolescents	Adults	Adolescents	Adults	Adolescents	Adults	Adolescents	Adults
% Never	17.4	20.6	15.2	14.7	14.2	12.3	14.4	14.6	17.0	21.6	12.8	17.5
% Rarely	16.0	20.6	26.5	23.1	25.8	21.5	26.0	19.9	35.0	16.6	31.2	24.1
% Sometimes	31.8	26.6	31.5	23.7	32.1	30.5	26.8	27.5	22.6	25.9	24.8	22.1
% Usually	24.6	22.9	20.2	28.4	21.9	26.8	28.8	28.8	18.7	25.6	22.8	23.4
% Always	10.2	9.3	6.6	10.0	6.0	8.9	4.0	9.2	6.7	10.3	8.4	12.9
Wear sunscreen	Adolescents	Adults	Adolescents	Adults	Adolescents	Adults	Adolescents	Adults	Adolescents	Adults	Adolescents	Adults
% Never	9.5	12.6	7.9	6.7	4.6	4.0	7.4	8.3	6.3	8.6	8.0	9.6
% Rarely	12.4	11.6	9.6	9.7	8.9	6.0	8.0	10.6	9.3	6.0	9.7	9.2
% Sometimes	22.0	19.6	22.8	12.4	19.2	20.5	21.4	15.9	23.0	18.6	21.8	17.8
% Usually	35.1	28.2	33.8	29.4	30.5	30.4	32.1	24.5	36.7	30.9	32.6	28.1
% Always	21.0	27.9	25.8	41.8	36.8	39.1	31.1	40.7	24.7	35.9	27.9	35.3
Wear a hat	Adolescents	Adults	Adolescents	Adults	Adolescents	Adults	Adolescents	Adults	Adolescents	Adults	Adolescents	Adults
% Never	19.0	19.9	15.6	19.0	12.6	11.2	16.0	12.9	17.0	12.9	16.1	13.5
% Rarely	17.7	10.6	18.5	7.4	16.2	12.6	12.4	13.5	20.0	11.0	20.8	12.5
% Sometimes	28.2	17.6	25.2	17.4	27.2	13.9	28.4	17.5	26.7	17.6	24.2	13.2
% Usually	20.0	24.3	26.5	25.8	30.8	29.8	27.8	24.2	26.7	28.9	23.8	26.7
% Always	15.1	27.6	14.2	30.4	13.2	32.5	15.4	31.8	9.6	29.6	15.1	34.0
Wear sunglasses	Adolescents	Adults	Adolescents	Adults	Adolescents	Adults	Adolescents	Adults	Adolescents	Adults	Adolescents	Adults
% Never	33.1	8.6	27.8	9.4	24.1	5.6	31.1	8.3	35.0	9.0	40.6	9.2
% Rarely	17.7	4.3	14.9	3.3	19.9	3.6	16.0	4.6	22.0	5.6	23.8	7.3
% Sometimes	14.4	9.6	24.5	8.4	15.6	8.9	18.1	8.3	16.7	10.0	14.8	8.9
% Usually	16.1	19.9	19.5	17.0	19.2	19.9	15.4	15.9	14.3	14.6	11.4	19.1
% Always	18.7	57.5	13.2	61.9	21.2	61.9	19.4	62.9	12.0	60.8	9.4	55.4
Seeking shade	Adolescents	Adults	Adolescents	Adults	Adolescents	Adults	Adolescents	Adults	Adolescents	Adults	Adolescents	Adults
% Never	2.3	1.3	3.0	4.3	0.7	1.0	2.7	4.0	2.7	4.3	1.7	5.3
% Rarely	10.1	10.6	12.9	10.0	8.2	7.3	15.7	5.6	6.3	6.0	11.1	9.6
% Sometimes	36.1	30.9	42.4	34.1	46.4	31.1	36.8	33.8	38.3	32.2	36.2	30.3
% Usually	44.6	41.9	38.1	40.1	39.1	46.7	38.1	41.0	44.3	46.2	44.3	42.6
% Always	6.9	15.3	3.6	11.4	5.6	13.9	6.7	15.6	8.3	11.3	6.7	12.2
Stay indoors	Adolescents	Adults	Adolescents	Adults	Adolescents	Adults	Adolescents	Adults	Adolescents	Adults	Adolescents	Adults
% Never	3.3	4.0	5.0	7.3	2.0	2.6	1.0	2.6	0.7	1.7	0.7	3.6
% Rarely	12.1	11.6	16.9	15.4	16.9	13.6	11.0	8.6	13.0	7.3	11.4	8.9
% Sometimes	39.0	40.9	42.3	32.4	40.1	40.7	40.5	33.8	36.3	35.2	36.5	33.0
% Usually	36.1	37.5	30.8	36.5	38.7	37.1	43.1	45.0	44.3	49.2	44.0	46.5
% Always	9.5	6.0	5.0	8.4	2.3	6.0	4.3	9.9	5.7	6.6	7.4	7.9

**Table 3:** Sample means of sun-related behaviours per survey year for adolescents and adults.

Variables	Survey Year							
**Adolescents (*N* = 1806)**	**2015/16**	**2016/17**	**2017/18**	**2018/19**	**2019/20**	**2020/21**	**χ^2^**	**φ’**
Wear protective clothing								
Mean (SD)	2.94 (1.23)	2.76 (1.14)	2.79 (1.12)	2.82 (1.12)	2.63 (1.16)	2.83 (1.17)	χ^2^ (20) = 52.88 ***	φ’ = 0.09 **
Wear sunscreen								
Mean (SD)	3.46 (1.22)	3.60 (1.20)	3.86 (1.15)	3.72 (1.20)	3.64 (1.14)	3.62 (1.21)	χ^2^ (20) = 29.20 *	φ’ = 0.06
Wear a hat								
Mean (SD)	2.94 (1.32)	3.05 (1.28)	3.16 (1.22)	3.14 (1.28)	2.92 (1.24)	3.01 (1.30)	χ^2^ (20) = 26.57 *	φ’ = 0.06
Wear sunglasses								
Mean (SD)	2.70 (1.53)	2.75 (1.39)	2.93 (1.49)	2.76 (1.51)	2.46 (1.40)	2.25 (1.34)	χ^2^ (20) = 67.47 ***	φ’ = 0.10 **
Seeking shade								
Mean (SD)	3.44 (0.85)	3.26 (0.84)	3.41 (0.75)	3.30 (0.91)	3.49 (0.84)	3.43 (0.84)	χ^2^ (20) = 37.01 *	φ’ = 0.07 *
Stay indoors								
Mean (SD)	3.36 (0.93)	3.14 (0.93)	3.23 (0.83)	3.39 (0.78)	3.41 (0.81)	3.46 (0.82)	χ^2^ (20) = 58.92 ***	φ’ = 0.09 **
**Adults (*N* = 1808)**	**2015/16**	**2016/17**	**2017/18**	**2018/19**	**2019/20**	**2020/21**	**χ^2^**	**φ’**
Wear protective clothing								
Mean (SD)	2.80 (1.26)	2.96 (1.23)	2.99 (1.16)	2.98 (1.20)	2.86 (1.30)	2.90 (1.30)	χ^2^ (20) = 28.74 *	φ’ = 0.06
Wear sunscreen								
Mean (SD)	3.47 (1.34)	3.90 (1.24)	3.95 (1.09)	3.79 (1.30)	3.79 (1.24)	3.70 (1.30)	χ^2^ (20) = 45.56 ***	φ’ = 0.08 **
Wear a hat								
Mean (SD)	3.29 (1.47)	3.41 (1.47)	3.60 (1.35)	3.48 (1.39)	3.51 (1.36)	3.55 (1.41)	χ^2^ (20) = 29.36 *	φ’ = 0.06
Wear sunglasses								
Mean (SD)	4.13 (1.27)	4.19 (1.28)	4.29 (1.13)	4.21 (1.27)	4.13 (1.32)	4.04 (1.33)	χ^2^ (20) = 17.26 *	φ’ = 0.05
Seeking shade								
Mean (SD)	3.59 (0.92)	3.44 (0.97)	3.65 (0.84)	3.59 (0.95)	3.54 (0.93)	3.47 (1.00)	χ^2^ (20) = 30.59 *	φ’ = 0.07
Stay indoors								
Mean (SD)	3.30 (0.90)	3.23 (1.05)	3.30 (0.87)	3.51 (0.88)	3.52 (0.79)	3.46 (0.90)	χ^2^ (20) = 52.23 ***	φ’ = 0.09 **

Note: SD = Standard deviation; χ^2^ = chi-squared test of independence; φ’ = effect size (Cramer’s V). * *p* < 0.05, ** *p* < 0.01, *** *p* < 0.001.

**Table 4:** Hierarchical linear regression results for adolescents and adults.

	Adolescents (*N* = 1806)			Adults (*N* = 1808)		
Variables	B (95% CI)	β	sr^2^	B (95% CI)	β	sr^2^
Step 1						
Age	0.02 (−0.01, 0.04)	0.03	0.00	0.03 (0.02, 0.03)	0.29 ***	0.09
Gender	0.03 (−0.03, 0.09)	0.02	0.00	0.01 (−0.05, 0.07)	0.01	0.00
Location	0.04 (−0.02, 0.11)	0.03	0.00	0.07 (0.00, 0.14)	0.05	0.00
Skin type	−0.11 (−0.14, −0.09)	−0.21 ***	0.04	−0.12 (−0.14, −0.09)	−0.18 ***	0.03
Step 2						
Age	0.02 (−0.01, 0.04)	0.03	0.00	0.03 (0.02, 0.03)	0.29 ***	0.09
Gender	0.03 (−0.03, 0.08)	0.02	0.00	0.01 (−0.05, 0.07)	0.01	0.00
Location	0.04 (−0.02, 0.11)	0.03	0.00	0.07 (0.00, 0.14)	0.05	0.00
Skin type	−0.11 (−0.14, −0.09)	−0.21 ***	0.04	−0.12 (−0.14, −0.09)	−0.18 ***	0.03
Year	−0.02 (−0.03, 0.00)	−0.04	0.00	0.01 (−0.01, 0.02)	0.01	0.00

Note. CI = Confidence interval. *** *p* < 0.001.

## Data Availability

Data can be made available upon request.
